# Impact of operational conditions on drinking water biofilm dynamics and coliform invasion potential

**DOI:** 10.1128/aem.00042-24

**Published:** 2024-04-22

**Authors:** Fien Waegenaar, Cristina García-Timermans, Josefien Van Landuyt, Bart De Gusseme, Nico Boon

**Affiliations:** 1 Department of Biotechnology, Center for Microbial Ecology and Technology (CMET), Ghent University, Ghent, Belgium; 2 Center for Advanced Process Technology for Urban Resource Recovery (CAPTURE), Ghent, Belgium; 3 Farys, Department R&D – Innovation Water, Ghent, Belgium; Washington University in St. Louis, St. Louis, Missouri, USA

**Keywords:** drinking water, distribution systems, biofilms, operational changes, coliforms, invasion

## Abstract

Biofilms within drinking water distribution systems serve as a habitat for drinking water microorganisms. However, biofilms can negatively impact drinking water quality by causing water discoloration and deterioration and can be a reservoir for unwanted microorganisms. In this study, we investigated whether indicator organisms for drinking water quality, such as coliforms, can settle in mature drinking water biofilms. Therefore, a biofilm monitor consisting of glass rings was used to grow and sample drinking water biofilms. Two mature drinking water biofilms were characterized by flow cytometry, ATP measurements, confocal laser scanning microscopy, and 16S rRNA sequencing. Biofilms developed under treated chlorinated surface water supply exhibited lower cell densities in comparison with biofilms resulting from treated groundwater. Overall, the phenotypic as well as the genotypic characteristics were significantly different between both biofilms. In addition, the response of the biofilm microbiome and possible biofilm detachment after minor water quality changes were investigated. Limited changes in pH and free chlorine addition, to simulate operational changes that are relevant for practice, were evaluated. It was shown that both biofilms remained resilient. Finally, mature biofilms were prone to invasion of the coliform, *Serratia fonticola*. After spiking low concentrations (i.e., ±100 cells/100 mL) of the coliform to the corresponding bulk water samples, the coliforms were able to attach and get established within the mature biofilms. These outcomes emphasize the need for continued research on biofilm detachment and its implications for water contamination in distribution networks.

**IMPORTANCE:**

The revelation that even low concentrations of coliforms can infiltrate into mature drinking water biofilms highlights a potential public health concern. Nowadays, the measurement of coliform bacteria is used as an indicator for fecal contamination and to control the effectiveness of disinfection processes and the cleanliness and integrity of distribution systems. In Flanders (Belgium), 533 out of 18,840 measurements exceeded the established norm for the coliform indicator parameter in 2021; however, the source of microbial contamination is mostly unknown. Here, we showed that mature biofilms, are susceptible to invasion of *Serratia fonticola*. These findings emphasize the importance of understanding and managing biofilms in drinking water distribution systems, not only for their potential to influence water quality, but also for their role in harboring and potentially disseminating pathogens. Further research into biofilm detachment, long-term responses to operational changes, and pathogen persistence within biofilms is crucial to inform strategies for safeguarding drinking water quality.

## INTRODUCTION

Microbial communities are ubiquitously present in drinking water distribution systems (DWDS). Over 98% of these microorganisms form biofilms on pipe materials or are associated with loose deposits (1–3). Biofilms can contribute to drinking water discoloration, the transformation of organic compounds, the decay of free chlorine, microbial regrowth, the formation of disinfection by-products, unwanted odor compounds, and so on (4–7). Additionally, they are recognized as potential sources of opportunistic pathogens (3, 8–12). Previous researchers detected pathogens such as *Mycobacteria* spp. and *Legionella* spp. as well as fecal indicators such as *Escherichia coli*, in biofilm samples from full-scale distribution networks (9, 11). Moreover, *Pseudomonas aeruginosa*, *Mycobacterium avium*, and *Legionella pneumophila* can persist in young biofilms after spiking 10^5^–10^6^ cells/mL (12, 13). As a result, biofilms are a potential risk to human health, as biofilm cells can be released to the planktonic water phase under certain conditions. To ensure the quality of drinking water, the measurement of coliform bacteria serves as an indicator for fecal contamination, playing a pivotal role in assessing the effectiveness of disinfection processes and the cleanliness and integrity of distribution systems (14, 15). In Flanders (Belgium), 533 out of 18,840 measurements exceeded the established norm for the coliform indicator parameter in 2021, with a maximum value of 201 coliforms per 100 mL (14). Similar maximum concentrations (e.g., 129 coliforms/100 mL or 175 coliforms/100 mL) have been measured before in other full-scale studies performed in the United States and Iran (16, 17). However, the source of microbial contamination is often not retrievable (14, 18).

Characterizing the microbial compositions and phenotypic attributes of biofilms, especially in the context of drinking water, poses challenges due to the practical difficulties in sampling. Previous researchers used laboratory setups to investigate the development and community compositions of biofilms (19–26). For example, biofilm formation rates, measured in terms of ATP activity, and the deposition of iron and manganese have been studied using biofilm monitors consisting of glass rings (27–29). Relatively new sampling techniques consist of a coupon holder to implement in a part of the DWDS or in pilot-scale networks (30, 31). Ginige et al. (28) showed that the microbial ATP content on glass rings was 80% less than that on plastic coupons for young biofilms. However, inert glass allows the bulk water characteristics to be the only variable determining biofilm formation and composition (27).

A typical drinking water biofilm comprises a diverse microbial community attached to distribution pipes and immersed in a self-produced matrix of extracellular polymeric substances (EPS), predominantly composed of polysaccharides and proteins (3, 32, 33). The bacterial biofilm community is dominated by *Proteobacteria*, more specifically *Alpha*- and *Gammaproteobacteria*. Notable genera found in biofilms on distribution pipes include *Pseudomonas*, *Sphingomonas*, and *Acinetobacter* (1, 6, 31, 34–42). The biofilm environment provides protection against various environmental challenges, including antibiotics, metals, disinfectants like free chlorine, and changes in operational conditions such as shear stress. This protection is attributed to the presence of the EPS matrix and the interconnected processes among the biofilm bacteria (43–45). The formation of biofilms, along with their corresponding phenotypic structure and the existing microbial community, is primarily influenced by the raw water source and treatment processes (e.g., microbial and nutrient composition) (3, 21, 46–49). For instance, previous studies reported a shift in the microbial biofilm community and concentrations on distribution pipes after switching from drinking water with more carbon and a higher conductivity to less turbid water with a lower nutrient content (47, 48). In addition, the composition of drinking water biofilms, and consequently biofilm detachment, is affected by factors such as water residence time, pipe materials, free chlorine concentrations, and temperature (5, 23, 25, 50–56). Former studies mainly focused on the bacterial removal effectiveness of hydrodynamic stressors such as flushing, increasing chlorination concentrations, or interrupted water flow (24, 51, 53, 56). For example, shock chlorination (10 mg Cl_2_/L, 60 min contact time) has been shown to remove 75% of the biofilm bacteria (24). However, there is a notable gap in the literature regarding the impact of minor operational variations, commonly encountered in practice, on the dispersal of biofilms into the water phase.

Here, two biofilm monitors, consisting of glass rings were set up to investigate whether unwanted coliforms can intrude into mature drinking water biofilms. First, the biofilms were characterized regarding bacterial cell density and community composition, as well as phenotypic parameters such as biovolume and thickness. Second, the response of the biofilm microbiome and possible biofilm detachment after minor water quality changes were investigated. These quality changes (pH, free chlorine concentration) were specifically chosen to simulate operational changes that are relevant to the full-scale DWDS. Finally, the survival of the coliforms in the bulk water phase was evaluated, and the attachment of these coliforms on the biofilm was investigated using confocal laser scanning microscopy (CLSM) and quantitative polymerase chain reaction (qPCR). We chose to spike coliforms in low concentrations (~100 cells/100 mL), in order to simulate real-life contaminations and thus conditions relevant for practice.

## MATERIALS AND METHODS

### Biofilm sampling device, conditions, and experimental design

A KIWA biofilm monitor was used (KWR, The Netherlands) consisting of 38 glass rings to collect drinking water biofilm (Figure A1) (27). This monitor was placed at two different locations for 17 months, receiving a continuous flow of 270 L/h, according to the manufacturer’s instructions. Monitor 1 was placed at the outlet of a drinking water reservoir receiving treated groundwater, before the UV post-disinfection step (Table A1). Monitor 2 was placed at the outlet of a drinking water tower receiving treated surface water with residual free chlorine. Water quality parameters were measured by the respective drinking water providers (57). The total organic carbon (TOC) concentration was measured at the end of the experiment (18). A timeline of the experiments conducted is represented in Table 1 and briefly described below.

**TABLE 1:** Experimental design detailing time points, specific experiments, water flow, number of rings analyzed, and types of analyses conducted^*a*^

Time point (months)	Place	Experiment	Water flow (L/h)	Rings taken for analysis	Type of analysis
1	Water tower/reservoir	Growing biofilm	270		
11	Water tower/reservoir	Growing biofilm	270	4	FCM, ATP, 16S
				3	CLSM (DAPI)
11	Water tower/reservoir	Growing biofilm	270	6	CLSM: test EPS staining
17	Water tower/reservoir	Growing biofilm	270	4	FCM, ATP, 16S
				3	CLSM (DAPI, EPS)
18	Lab	Changing conditions: LSI = 0.30	42	Hour 0: 2	FCM
18	Lab	Changing conditions: LSI = −0.50	42	Hour 0: 2	FCM
19	Lab	Changing conditions: HOCl addition	42	Hour 0: 2	FCM
20	Lab	Invasion (first experiment)	42	Day 0: 2	FCM, qPCR, CLSM (DAPI)
				Day 4: 2	FCM, qPCR, CLSM (DAPI)
				Day 7: 1	CLSM (DAPI)
24	Lab	Invasion (second experiment)	42	Day 0: 2	FCM, qPCR CLSM (DAPI)
				Day 8: 1	qPCR, CLSM (DAPI)

^*a*^ To perform FCM, ATP, 16S sequencing, and qPCR, a ring was placed in 10 mL of autoclaved tap water and subjected to sonication (three cycles of 2 min each) to detach the biofilm.

After 11 and 17 months, three rings were analyzed with CLSM. Four rings were each put in 10 mL autoclaved tap water and sonicated in a water bath (37 kHz, Elma – Ultrasonic, Belgium) three times for 2 min with a vortex step in between according to the manufacturer’s instructions, to detach the biofilm (see supplementary methods for details). The biomass was quantified in terms of ATP and total cell counts (TCC) and the microbial community was characterized with 16S rRNA amplicon sequencing as described further. In the second part of the study, the KIWA monitors were transported to laboratory conditions to be able to manipulate the water conditions and to perform invasion experiments on the 17 months old biofilms. During these experiments, we utilized the same types of water (i.e., treated groundwater and treated surface water) as those employed in the preceding experiments (Table A1). The monitor was connected to a 10 L plastic vessel and the water was pumped (WM 323 peristaltic pump, Watson Marlow, Belgium) and recirculated over the biofilm monitor. The pump was operating at 150 rpm and the flow was 42 L/h. The experiments were performed at room temperature (i.e., 22 ± 2°C). As the biofilms were consistently exposed to varied experimental conditions, flow cytometry (FC) was conducted on a ring before each experiment to define the total cell density (Table A2).

### Changing operational conditions in terms of the Langulier Saturation Index and HOCl addition

The LSI was calculated according to WAC/III/A/011 (58) (Table 1). The pH, conductivity, and calcium concentration were measured using a Multi-parameter analyzer C1010 (Consort, Belgium), a Hanna Edge Conductivity Meter (HANNA Instruments, Belgium), and the Total Hardness Test (Merck, Belgium), respectively. The alkalinity of the water samples was determined based on WAC/III/A/006 (59). Using 1M NaOH and 1M HCl (Chem-lab, Belgium), the pH was adapted to the higher and lower Langelier Saturation Index (LSI) (Table 2). In addition, HOCl solution was added to have a free chlorine concentration of 0.20 and 0.28 mg/L, for the groundwater and surface water samples, respectively. The chlorine concentrate solution was prepared by the addition of a NaOCl tablet (B-Care Chemicals, Belgium) to 1 L ultrapure water (Milli-Q, Merck Millipore, Germany). The amount of free chlorine was quantified with the Pocket Colorimeter II (Hach, Belgium). Samples of the bulk water were taken every 2 h over a period of 8 h to measure with flow cytometry.

**TABLE 2:** Original pH and corresponding Langelier Saturation Index (LSI) for each water type^*a*^

Water type	pH original	LSI original	pH, HCL added	LSI, HCl added	pH, NaOH added	LSI, NaOH added
Treated groundwater (no disinfection)	7.10	−0.04	6.84	−0.52	7.58	0.25
Treated surface water (chlorination)	7.75	0.10	7.30	−0.55	7.90	0.31

^*a*^ To evaluate biofilm detachment, the LSI was changed by decreasing or increasing the pH using HCl and NaOH, respectively.

### Invasion experiments with GFP-expressing *Serratia fonticola*

The strain was isolated from the Flemish drinking water distribution network (Antwerp, Pidpa) and identified with MALDI-TOF mass spectrometry using a Vitek MS (bioMérieux, Marcy-l'Étoile, France) and 16S rRNA gene Sanger sequencing as described by Kerckhof et al. (60) and identified using the NCBI BLAST tool (61). The strain was made ampicillin resistant by serial selections on Luria Broth (LB) agar (Carl Roth, Belgium) with increasing concentrations of ampicillin (5–100 µL/mL) (Merck, Belgium). Each time, the cultures were incubated for 24 h at 28°C. The strain was genetically engineered to express the GFP protein using triparental mating. Briefly, the donor strain *E. coli* DH5α pME6012 containing the plasmid ptac-gfp and the helper strain *E. coli* HB101 containing the plasmid pRK2013 needed for conjugation were grown in LB medium at 37°C for 24 h with 8 µg/mL tetracycline and 50 µg/mL kanamycin, respectively. The acceptor, *Serratia fonticola*, was grown in LB medium with 50 µg/mL ampicillin at 28°C for 24 h. Next, the cultures were washed two times. They were centrifuged for 5 min at 3,000 × *g*, the supernatant was removed and 0.22 µm filtered sterile phosphate-buffered saline (PBS) (PBS tablet, Merck, Belgium) was added. Triparental mating was performed by adding 10 µL of each culture on LB agar. Plates were incubated at 28°C for 24 h. GFP-expressing coliforms were selected for resistance to tetracycline (8 µg/mL) and ampicillin (50 µg/mL) and screened for GFP fluorescence using a dark reader (Clare Chemical Research, USA). The final identification of the pure culture was performed with 16S rRNA gene Sanger sequencing as described in Kerckhof et al. (60) and identified using the NCBI BLAST tool (61). The strain is available in the Belgian Coordinated Collection of Microorganisms under collection number LMBP 13927.

Two invasion experiments were performed by spiking GFP-expressing *S. fonticola* to the corresponding water samples (*V* = 10 L) in plastic vessels that were connected to the KIWA monitor. Therefore, a few colonies were picked from the agar plate and resuspended in 25 mL of 3 g/L sterile R2A broth medium (Oxoid, England) with tetracycline (8 µg/mL) and ampicillin (50 µg/mL) and the tubes were incubated at 28°C and 100 rpm for 24 h. Subsequently, the culture was washed using sterile 8.5% NaCl as explained before. Afterward, the culture was transferred to a diluted liquid medium of 50 mg/L R2A broth medium (Oxoid, England) with tetracycline (8 µg/mL) and ampicillin (50 µg/mL) and incubated at 28°C and 100 rpm for 24 h. The culture was washed before measuring the TCC with flow cytometry. The culture was diluted using sterile 8.5% NaCl and final spike concentrations were ranging from 50 to 500 cells/100 mL. The concentration of *S. fonticola* in the bulk water was determined by filtering (3 × 100 mL) on S-Pack filters 0.45 µm (Merck, Belgium) using a filtration unit consisting of six filtration funnels and a Microsart e.jet vacuum pump (Sartorius, Germany) and incubation (18–23 h, 37°C) on chromogenic coliform agar (CCA) (Carl Roth, Belgium), according to the ISO 9308-1:2014 method for drinking water (57). As a control measure, the bulk water was filtered for selective plating and a ring was extracted for FC and qPCR analysis, as well as for CLSM analysis, preceding each invasion experiment (Table 1). Water was refreshed after 4 days of recirculation.

### Confocal laser scanning microscopy

Visual characterization of the biofilms was done with a Nikon A1R confocal microscope (Nikon Instruments Inc., USA), which consists of four lasers with in total seven laser lines (405, 457, 476, 488, 514, 561, and 639 nm). After 11 months, three biofilm rings were taken for CLSM analysis (Table 1). Prior to analysis, samples were fixed in 4% paraformaldehyde (Merck, Belgium) for 24 h. Fixated biofilm rings were stored at −20°C in a PBS:EtOH 1:1 solution prior to analysis. First, samples were dehydrated in an increasing ethanol series [3 min each in 50%, 80%, and 96% (vol/vol) ethanol]. Second, nucleic acids were labeled with 3 µM DAPI [Excitation/Emission (Ex/Em): 352/464, Merck, Belgium] for 20 min in the dark at room temperature. Samples were washed with cold (i.e., ±6°C), filtered through 0.2 µm, PBS and air-dried. Finally, Fluoroshield mounting medium (Merck, Belgium) was added to prevent bleaching. On each ring (*n* = 3), one stack (±30 images) of horizontal plane pictures (10×/0.30 air objective, 512  ×  512 pixels equivalent to 1,282.58  ×  1,282.58 µm^2^) with a z-step of 4.8 µm was taken at three locations (randomly selected) of each biofilm ring. After 17 months, an additional set of three rings was employed for CLSM analysis, this time incorporating EPS staining. Fixation, storage, and dehydration were conducted the same way as previously described. Then, the protein content was stained for 30 min with FilmTracer Sypro Ruby Biofilm Matrix Stain (Ex/Em: 450/610, ThermoFisher Scientific, Belgium) and the carbohydrates with 240 µM Concanavalin A CF640R (Ex/Em: 642/663, Biotium, USA) for 30 min. Nucleic acids were labeled with 3 µM DAPI (Ex/Em: 352/464, Merck, Belgium) for 20 min. Staining was performed at room temperature (i.e., ±22°C) and in the dark, after each staining step a washing step with cold (i.e., ±6°C), filtered through 0.2 µm, PBS. Finally, the rings were air-dried and Fluoroshield mounting medium (Merck, Belgium) was added to prevent bleaching. The selection of these fluorophore pairs was based on studies conducted by Fish et al. (62) and Birarda et al. (63). However, as noted by Birarda et al. (63), it is acknowledged that Concanavalin A can bind to glycoproteins and glycolipids. Nonetheless, the abundance of biofilm matrix polysaccharides is presumed to surpass that of these two components, making them the primary contributors to positive carbohydrate staining. On each ring (*n* = 3), one stack (±30 images) of horizontal plane pictures (10×/0.30 air objective, 1,024  ×  1,024 pixels equivalent to 1,272.79  ×  1,272.79 µm^2^) with a z-step of 4.8  µm was taken at three locations (randomly selected) of the ring. For evaluating invasion of the GFP-expressing coliform (Ex/Em: 488/509), each time a ring before and after were analyzed (Table 1). Fixation, storage, dehydration and DAPI staining were conducted the same way as previously described. On each ring (*n* = 1), one stack (±30 images or ±20 images) of horizontal plane pictures (10×/0.30 air objective, 1,024  ×  1,024 pixels equivalent to 1,272.79  ×  1,272.79  µm or 40×/0.60 air objective, 1,024  ×  1,024 pixels equivalent to 202.42  ×  202.42 µm^2^) with a z-step of 2 µm was taken at five locations (randomly selected) of the ring. All samples were analyzed within 14 days. A construction was made and Nunc Glass Bottom Dishes (ThermoFisher Scientific, Belgium) were used to fit the biofilm ring under the CLSM. Image stacks were processed in ImageJ and the plugin Comstat2 was used to determine biovolume, roughness, and thickness of the biofilms (64). An automatic threshold (Otsu’s method) was used for all image processing. Particles stained by DAPI are further reported as DAPI-stained cells and/or DAPI-stained biomass.

### Flow cytometry and ATP analysis

ATP and TCC were determined as described in Waegenaar et al. (18). The ATP concentration was measured using the BacTiter-Glo Microbial Cell Viability Assay (Promega, Belgium) and luminescence was measured with the Infinite M Plex, multimode microplate reader (Tecan, Switzerland). TCC was measured using an Attune NxT BRXX flow cytometer (ThermoFisher Scientific, USA) and staining was performed with 1 vol% of 100 times diluted SYBR Green I solution (10,000× concentrate in DMSO, Invitrogen, Belgium). Biofilm samples were 10 times diluted in 0.2 µm filtered bottled water (Evian, France) and all samples were measured in technical triplicate.

### Molecular analysis of microbial communities

16S rRNA gene amplicon sequencing was performed on biofilm and bulk samples. Biofilm samples (volume = 15 mL) were filtered using Millipore Express PLUS Membranes (Merck, Belgium) and Polycarbonate syringe filter holder (Sartorius, Germany). MF-Millipore Membrane Filters (Merck, Belgium) and a filtration unit consisting of six filtration funnels and a Microsart e.jet vacuum pump (Sartorius, Germany) were used to filter bulk water samples. DNA extraction was performed using the DNeasy PowerSoilPro kit (Qiagen, Germany), following the manufacturer’s protocol. PCR amplification was performed according to Van Landuyt et al. (65) (see supplementary methods for details). About 10 µL genomic DNA extract was sent out to LGC Genomics GmbH (Berlin, Germany) for library preparation and sequencing on an Illumina Miseq platform with v3 chemistry (Illumina, USA).

### Quantitative polymerase chain reaction to detect *Serratia fonticola*

QPCR assays were performed using a StepOnePlus real-time PCR system (Bio-Rad, Belgium). Specific primers were used to detect the gfp-gen: forward (5′- AGTGGAGAGGGTGAAGGTGA-3′) and reverse (5′-ACGGGAAAAGCATTGAACAC-3′). Reactions were performed in a volume of 20 µL consisting of 10 µL of 2× iTAQ universal SYBR Green supermix (Bio-Rad Laboratories, USA), 2.0 µL DNA template, 0.8 µL (10 µM) of each primer, and 6.4 µL nuclease-free water. Amplification conditions were outlined according to the manufacturer’s instructions. Quantification was done a standard curve based on known concentrations of DNA standard dilutions from 10^7^ to 10 copies/µL. The reactions were performed using undiluted and 10-fold diluted samples in technical triplicates, with both negative and positive controls included. The amplification plots, plateau phases, and the cycle threshold efficiencies were evaluated to ascertain the presence of detection signals and to determine the optimal sample dilution for positive detection (Figure A5).

### Data analysis and statistics

Data analysis was done in R (66) in RStudio version 4.2.1 (67). The Flow Cytometry Standard (.fcs) files were imported using the flowCore package (v2.12.2) (68). The background data were removed by manually drawing a gate on the FL1-H (green) and FL3-H (red) fluorescence channels as described in Props et al. (69). The xlsx package (v4.2.5.2) was used to analyze the data from the confocal microscopy (70). Illumina data were processed using the DADA2 pipeline (v1.28.0) (71). Taxonomy was assigned using the Silva database v138 (72). Further data analysis was performed using statistical packages such as the phyloseq package (v1.44.0) and the vegan package (v2.6-4) (73, 74). Data visualization was done using the ggplot2 (v3.4.3) and ggpubr (v0.6.0) packages (75, 76). The data generated by MALDI-TOF mass spectrometry was analyzed using the MYLA software. Statistical analysis was done with the dplyr package (v1.1.2) and the vegan package (v2.6-4) (74, 77).

## RESULTS

### Mature biofilm characterization

A KIWA biofilm monitor was used to grow and sample a drinking water biofilm (Figure A1). The monitor was set up at two distinct locations, each receiving water from different sources: treated groundwater without a disinfection step and treated surface water with a chlorination step. The treated surface water was characterized by elevated mineral content, including aluminum, calcium, and nitrate, leading to increased hardness. In contrast, treated groundwater exhibited a higher total organic carbon content and more total colony counts (Table A1).

**Fig 1:**
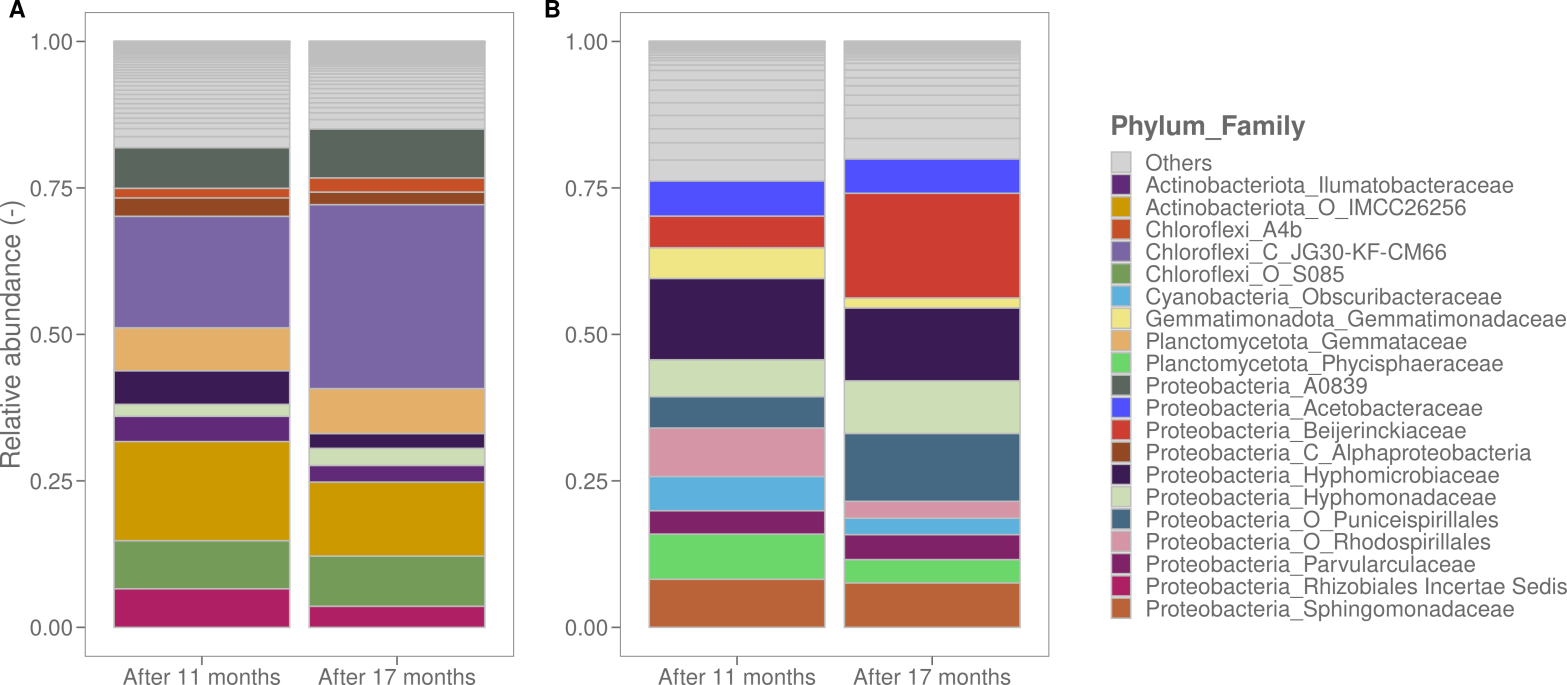
Relative abundances of the 20 most abundant families from the treated groundwater (not disinfected) (**A**) and treated surface water (chlorinated) (**B**) biofilms. Biofilm samples (*n* = 2) were taken after 11 and 17 months. Significant differences were observed between both biofilms at each time point (ANOSIM, *P* < 0.05).

After 11 and 17 months of biofilm development, we characterized the biofilms through flow cytometry, ATP analysis, CLSM, and 16S rRNA sequencing (Tables 1 and 3; Fig. 1). The cell densities and ATP concentrations of the groundwater biofilms were 10 times higher than those of the surface water biofilms. Similar observations were made using CLSM, where DAPI staining was used to determine the biofilm biomass, roughness, and average thickness. The biofilm derived from treated surface water exhibited increased roughness but decreased average thickness both at the 11-month and 17-month intervals. Notably, the treated groundwater biofilms exhibited reduced roughness and average thickness after 17 months compared to the measurements performed after 11 months, possibly indicating more compact biofilms. In general, significant statistical differences were observed between the two biofilms for each parameter after growing the respective biofilms for 17 months (Table 3). In addition, using 16S rRNA sequencing, a significant difference in the compositions of the two biofilm communities was observed (ANOSIM, *P* < 0.05, Fig. 1). *Chloroflexi* and *Proteobacteria* were identified as the most dominant phyla in the groundwater biofilm samples, constituting 37% and 35% of the community, respectively. *Chloroflexi* were mainly represented by uncultured and unclassified JG30-KF-CM66 (~30%) and S085 (~7%) bacteria, whereas only 1.5% of the groundwater bulk bacteria belonged to this phylum. The bulk community was dominated by *Cyanobacteria* (~26%) and *Alphaproteobacteria* (~22%), including families like *Hyphomicrobiaceae* (~3%) and *Hyphomonodaceae* (~3%) (Figure A3). These families were also detected in the surface water biofilm samples (i.e., ~3% and ~11%, respectively) and to a lesser extent in the corresponding bulk samples (i.e., ~2% and ~7%, respectively). More than 65% of the bacteria in the biofilms developed under surface water supply were *Alphaproteobacteria*, more specifically *Acetobacteraceae* (~12%), *Beijerinckiaceae* (~8.5%), and *Sphingomonadaceae* (~ 7%). Furthermore, these bacteria were predominant in the surface water bulk samples (~70%, Figure A3). To conclude, the phenotypic and genotypic characteristics were significantly different between biofilms derived from treated groundwater and treated surface water.

**TABLE 3:** Characterization of the biofilms using different techniques after 11 and 17 months^*a*^

Technique	Parameter	After 11 months		*P* value	After 17 months		*P* value
	Water type	Treated groundwater (no disinfection)	Treated surface water (chlorinated)		Treated groundwater (no disinfection)	Treated surface water (chlorinated)	
Flow cytometry	Total cell counts (cells/cm²)	(1.01 ± 0.04) × 10^7^	(5.96 ± 1.01) × 10^5^	4.11 × 10^−5^	(7.46 ± 0.58) × 10^6^	(5.23 ± 3.68) × 10^5^	4.11 × 10^−5^
ATP analysis	ATP content (pg/cm²)	411.34 ± 52.58	51.86 ± 3.22	8.23 × 10^−5^	896.54 ± 72.11	77.26 ± 41.48	4.04 × 10^−4^
CLSM	Roughness (Ra)	1.25 ± 0.39	1.97 ± 0.03	0.06	0.78 ± 0.14	1.68 ± 0.04	3.97 × 10^−5^
CLSM	Average thickness (µm)	15.78 ± 5.89	0.94 ± 0.57	0.06	4.95 ± 2.62	1.31 ± 0.33	7.18 × 10^−3^
CLSM	DAPI-stained biomass (µm³/µm²)	5.65 ± 2.23	0.57 ± 0.45	0.08^*t*^	4.10 ± 3.07	0.25 ± 0.10	1.59 × 10^−4^
CLSM EPS	Protein biovolume (µm³/µm²)	Not determined	Not determined		1.23 ± 1.09	0.13 ± 0.07	3.97 × 10^−5^
CLSM EPS	Sugar biovolume (µm³/µm²)	Not determined	Not determined		2.73 ± 2.54	0.29 ± 0.25	7.94 × 10^−5^

^*a*^ Results are presented as an average ± standard deviation. Statistics are done using the Wilcoxon rank-sum test or the *t* test indicated with a ‘*t*’, and statistical significance is considered when the *P* value < 0.05. Biofilms resulting from treated groundwater were more dens and active than biofilms resulting treated chlorinated surface water.

**Fig 2:**
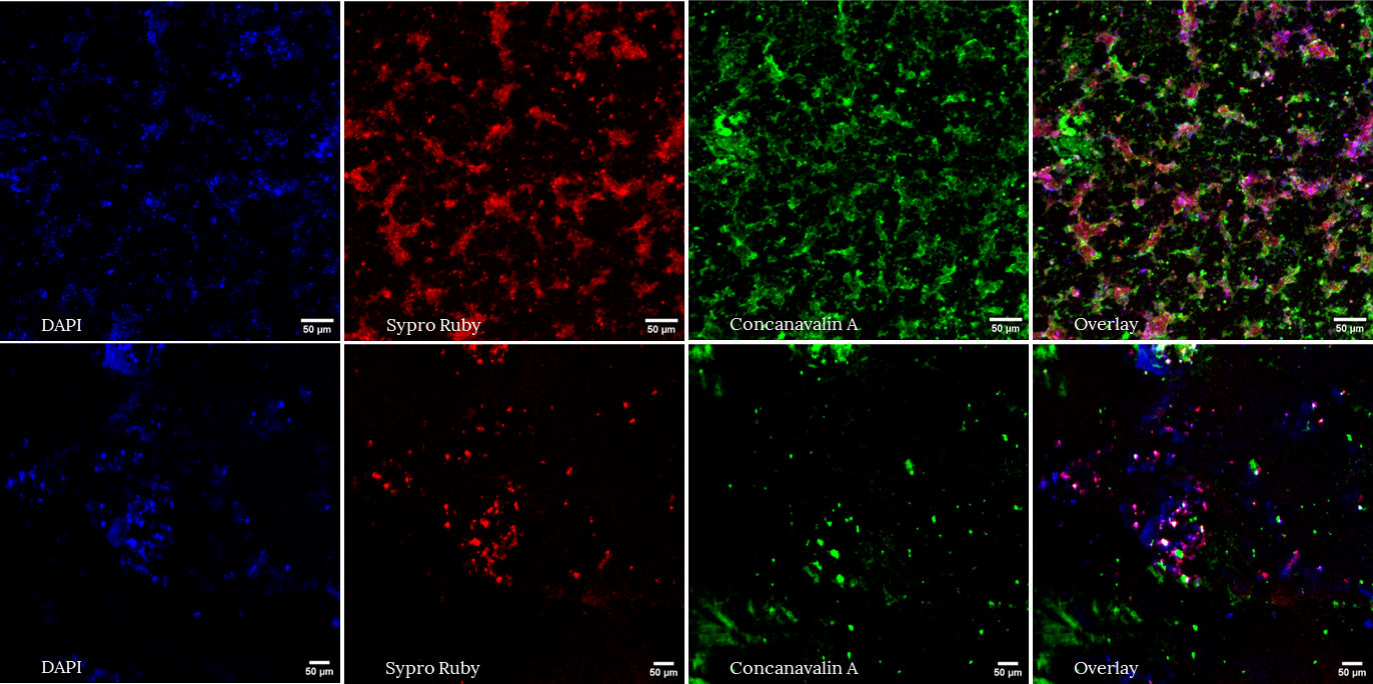
Confocal microscopy images of the treated groundwater (top images) and treated surface water (bottom images) biofilms. Biofilm cells were stained using DAPI and the EPS content, more specifically the protein and sugar content were stained with FilmTracer sypro ruby biofilm matrix stain and concanavalin A, respectively. Samples were analyzed after 17 months and the images were processed using ImageJ.

As EPS play a crucial role in the biofilm structure, the protein and sugar content were determined for both biofilm samples after 17 months (Tables 1 and 3; Fig. 2). Consistent with the other measurements, the groundwater biofilm exhibited 10 times more EPS biovolume than the surface water biofilm. However, the ratios of sugars to biomass and proteins to DAPI-stained biomass were similar for both mature biofilms (Figure A4). The higher bacterial and EPS content in the groundwater biofilm could be attributed to the higher carbon content in the raw water and the absence of disinfectants (Table A1). To illustrate that the biofilms used in further experiments were mature, statistical analyses were performed between the two time points, more precisely after 11 and 17 months (Table A3). DAPI-stained biomass and community composition were selected as key parameters, and no significant differences were observed over time for both biofilms.

### Effect of operational water quality changes on biofilm detachment

**Fig 3:**
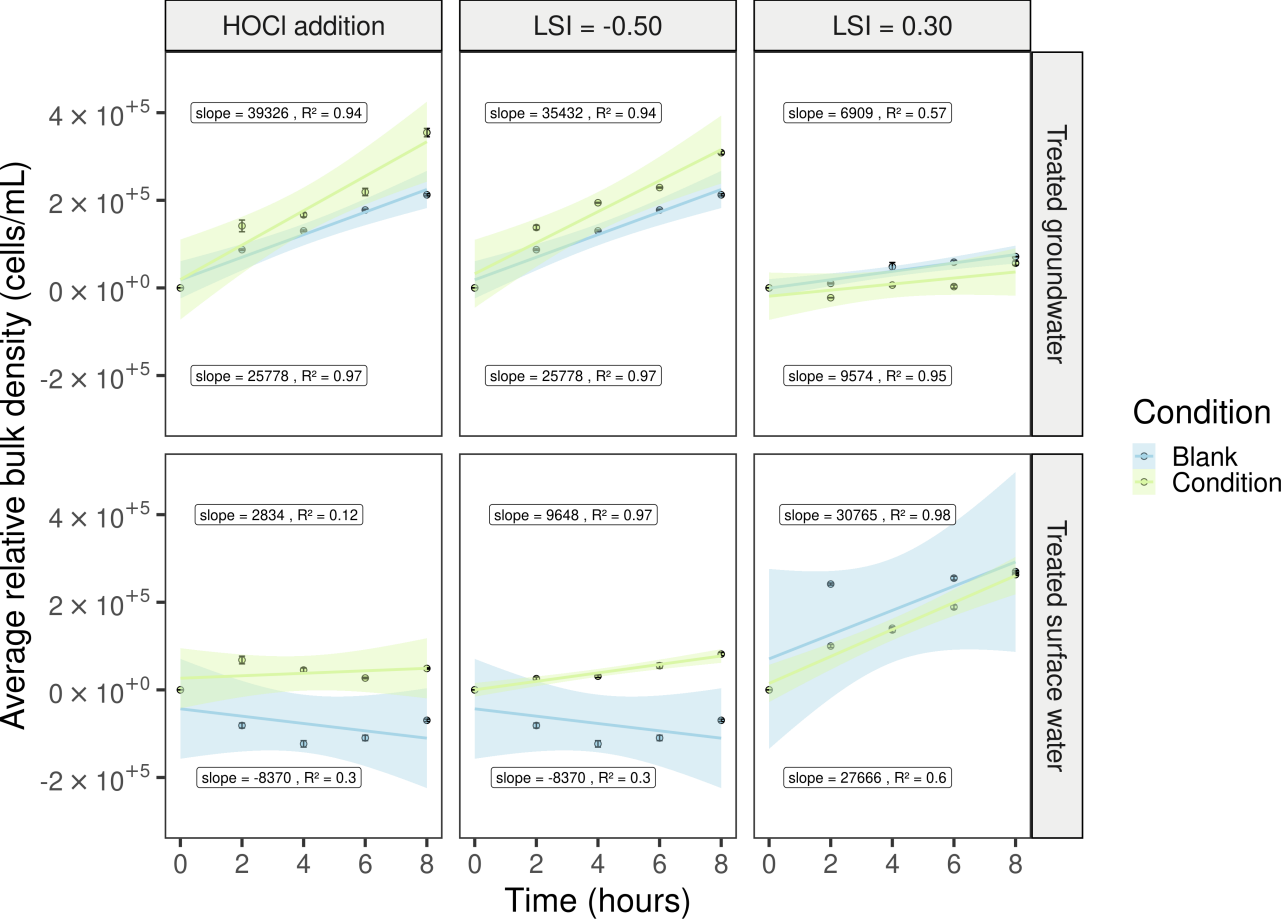
Average cell density (density_timepointx_ − density_timepoint0_) of the bulk water phase (*n* = 3) in function of time (hours) for each water type. The reference detachment profile is indicated with blue, the water cell density after applying different water quality changes (free chlorine, pH) is indicated with green. A linear regression model was calculated (95% CI) and the corresponding slopes and *R*^2^ are shown in a black box.

After 17 months of growing a drinking water biofilm, the KIWA monitors were transferred from the water reservoirs to laboratory conditions, and the original water quality was adjusted to examine the response of the biofilm microbiome to minor changes observed in practice (Table 1). More concretely, the effects of slight pH variations and the addition of small concentrations of HOCl on biofilm detachment were investigated. These minor pH changes led to an increase or decrease in the LSI (Table 2). This qualitative index predicts the scale-forming potential of water and is based on the measurement of pH, conductivity, alkalinity, calcium ions, and temperature (78, 79). Cell counts in the bulk were measured with flow cytometry for 8 h. Generally, little or no detachment of the biofilm was observed compared to the untreated controls (Fig. 3). Linear regression with a confidence interval of 95% was performed to quantify cell release rates to the bulk. If the cell release rate is higher than zero, there is an indication that biofilm cells are dispersed into the bulk water phase. Briefly, the detachment was always observed except for two blank conditions. Furthermore, the dispersal of biofilm cells to the bulk water was faster (i.e., higher release rates) when the LSI was changed or when HOCl was added. However, statistical analysis of the residuals and slopes of the linear regression models was conducted, and no significant difference was observed between the controls and the applied operational changes (Table A4). This implies that both biofilms remained resilient.

### Invasion of coliforms onto drinking water biofilms

**Fig 4:**
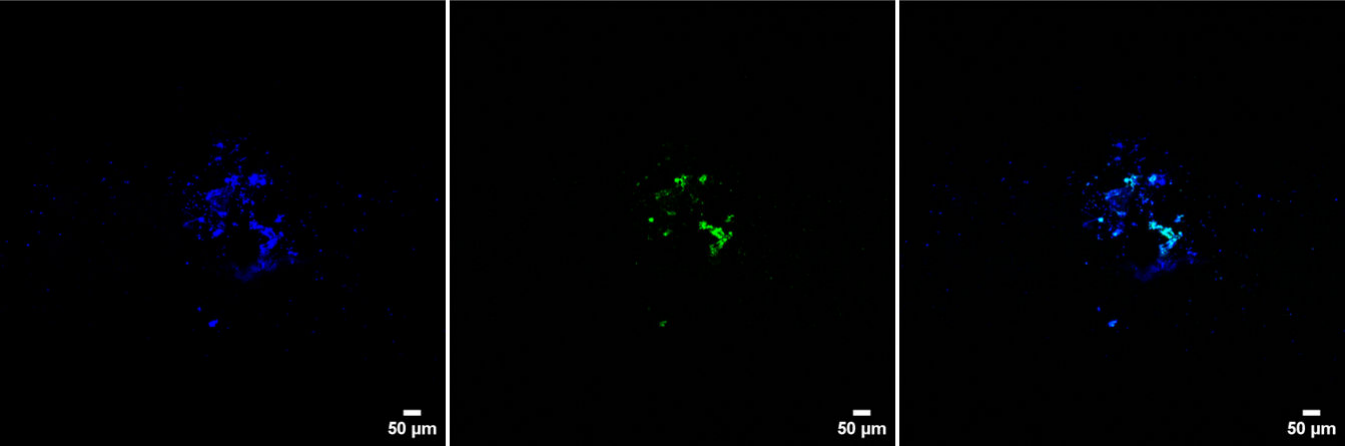
Confocal microscopy images (10×/0.3 air objective) of the first invasion experiment with *Serratia fonticola* for the treated surface water biofilm (8 days after the spike). From left to right: biofilms stained with DAPI, the GFP-expressing coliforms, an overlay image.

*S. fonticola*, isolated from the Flemish distribution network (Belgium), was genetically engineered to express the GFP protein, making it fluorescent and distinguishable from other drinking water biofilm bacteria. It was then introduced into the water supplied to the biofilm monitors. Two invasion experiments were performed for each water type (Table 1). The initial concentration and survival of the coliform in the bulk water were monitored using selective media. QPCR and CLSM were used to determine whether *S. fonticola* was attached to the biofilm (Table 1). Prior to conducting the invasion experiments, control measures were executed for bulk and biofilm samples, and no coliforms were detected. *S. fonticola* was added to the water samples and concentrations were ranging between 15 cells/100 mL and 650 cells/100 mL (Table 4). For treated surface water, the coliforms were still present in the bulk water phase after 3 h. After 24 h, the coliforms were below detection limit (<0 cells/100 mL) for both water types. Biofilms on the glass rings were analyzed after 4 or 7 days using CLSM and qPCR. For the first invasion experiment, the gene copies were below the limit of quantification (LOQ = 28.57 gene copies/cm²), whereas for the second invasion experiment, 5,676.46 ± 375.61 and 358.36 ± 24.03 gene copies/cm² of the invader were measured in the groundwater and surface water biofilms, respectively (Table 4, Figure A5). Detection of the GFP-expressing *S. fonticola* in the biofilm was also done with CLSM and coliforms were detected 7 or 8 days after the spike for each water type (Fig. 4; Figures A6–A13). Even though mature biofilms seem to be strong microbial ecosystems and minor water quality changes do not lead to major detachment into the bulk phase, they are susceptible to unwanted invasion by coliforms.

**TABLE 4:** Two invasion experiments were performed with *Serratia fonticola* on the mature biofilms^*a*^

		Bulk			Biofilm	
Technique	Experiment	Chromogenic coliform agar			CLSM	qPCR
Water type		Start concentration (cells/100 mL)	After 3 h (cells/100 mL)	After 24 h (cells/100 mL)	(-)	(gene copies/cm²)
Treated groundwater (no disinfection)	First invasion experiment	104.0 ± 10.6	0	0	D4: not present D8: present	D4: under LOQ
Treated groundwater (no disinfection)	Second invasion experiment	15.3 ± 3.0	0	0	D7: not possible	D7: 5,676.46 ± 375.61
Treated surface water (chlorinated)	First invasion experiment	650.0 ± 14.1	420.0 ± 17.0	0	D4: not present D8: present	D4: under LOQ
Treated surface water (chlorinated)	Second invasion experiment	39.5 ± 5.0	1.0 ± 1.4	0	D7: present	D7: 358.36 ± 24.04

^*a*^ The concentration in the bulk water phase was followed with selective plating (chromogenic coliform agar). Invasion of the coliform in the biofilm was determined using CLSM and qPCR. Positive detection on the respective day after the spike (Dx) with CLSM is indicated with “present.” It was not possible to perform CLSM for the groundwater biofilms of the second spike experiment because of calcium precipitation on the rings. Using qPCR, the GFP-expressing coliform was found in the biofilm after 7 days (D7).

## DISCUSSION

### Treated groundwater and chlorinated surface water resulted in significantly different biofilms in terms of community compositions and biomass content

Two biofilm monitors, consisting of glass rings, were employed to investigate drinking water biofilms. They were placed in two distinct reservoirs for 17 months, one receiving treated groundwater without disinfection and the other receiving treated surface water with residual free chlorine. The use of glass as a carrier material ensures reproducible biofilm sampling, considering only the biofilm formation potential. However, due to the absence of nutrient leaching, glass results in lower biofilm density compared to plastic (28). In general, bacterial cell densities of mature biofilms vary from 10^4^ to 10^8^ cells/cm² (2, 3, 22, 80). We observed similar concentrations, although a lower cell density was observed for biofilms developed under surface water supply, probably due to a free chlorine disinfectant (Table 3). Furthermore, the results aligned with previous studies, showing ATP concentrations of 100–1,100 pg/cm² for unchlorinated and 10–100 pg ATP/cm² for chlorinated water (38, 81). Regarding CLSM analysis results, lower DAPI-stained biomass content was observed for treated surface water biofilms, hovering around 1 µm³/µm², as previously reported in the literature (5). A study by Shen et al. (82) demonstrated a positive correlation between biofilm roughness and adhesion and a negative correlation with detachment. Although significantly higher biofilm roughness was measured for the surface water biofilm, this did not result in higher biomass content. Overall, biofilms from treated groundwater exhibited 10 times higher FC cell concentration, biofilm thickness, ATP, and DAPI-stained biomass content, possibly due to higher cell and TOC concentrations in bulk water and the absence of disinfection (Table 3; Table A1). Furthermore, chlorination not only reduced biofilm density but also decreased EPS production (Fig. 2; Table 3) (22). 16S rRNA sequencing revealed a significant difference between treated groundwater and surface water biofilms (Fig. 1). The groundwater biofilm community was dominated by *Alphaproteobacteria*, *Chloroflexi*, and *Actinobacteriota*, while the surface water biofilm mainly consisted of *Alphaproteobacteria* and *Gemmatimonadota*. Within the *Chloroflexi* phylum, the class JG30-KF-CM66 was most abundant, even though only small concentrations were found in the bulk. This finding is consistent with raw groundwater measurements reported in the literature (Fig. 1, Figure A3) (83). Additionally, members of the *Chloroflexi* and *Actinobacteriota* clusters are known to degrade complex organic matter structures that could be present in biofilms (84, 85). On the other hand, previous studies have confirmed that the biofilm and bulk core microbiome of treated chlorinated surface water mainly consists of *Alphaproteobacteria*, such as *Hyphomicrobiaceae* and *Sphingomonadaceae* (20, 26, 42, 54, 86–88). These taxa are both known to easily colonize surfaces, produce EPS, and can degrade a wide range of organic carbon compounds (20, 87, 89).

This study demonstrated that the biofilms entered a mature phase, also referred to as quasi-steady state phase, after 17 months, due to a consistent biofilm density and community during the 11-month to 17-month intervals (Table A3). Previous studies have indicated that this mature phase is characterized by stable cell numbers, EPS formation, and maintaining an equilibrium between growth, attachment, and detachment (40, 80). Furthermore, we showed that treated groundwater and chlorinated surface water resulted in significantly different biofilms, impacting cell density, and community composition. This underscores the importance of both water source and treatment processes in biofilm formation. However, conflicting findings persist regarding the impact of source and treatment in distribution pipes. Previous studies have suggested that the source water mainly shapes the biofilm community composition (36, 41). In contrast, other researchers have shown that there is no significant difference in the biofilm community concerning the drinking water source, and that treatment (e.g., disinfection) is more important for the biofilm core community in distribution pipes (90).

### Mature biofilms react minimally toward operational changes in water quality

While the water quality in drinking water distribution systems generally remains constant, minor operational adjustments can occur, impacting water microbiology (91). For example, additional chlorination because of water works, variations in the quality of raw water sources, mixing of different water types in the DWDS, and so on (91, 92). Previous studies have primarily focused on the influence of severe operational changes, such as flushing and shock chlorination, the goal of this work is to investigate the effects of these minor variations, which frequently occur in practice, on biofilm detachment.

Drinking water providers maintain fixed pH values and free chlorine dosage during treatment to control biofilm formation and corrosion of pipe materials, valves, pumps, and so on (15). To evaluate water corrosivity, the LSI, a qualitative index that predicts the scale forming potential of water, is determined. Both European and Belgian drinking water directives recommend measuring an LSI above −0.5 (14, 93). A slightly negative LSI means more corrosive water that contains carbon dioxide deposits, while a positive LSI indicates CaCO_3_ supersaturated water with the potential to form scale (78). In this study, the LSI of the tested waters was adjusted by changing the pH (Table 2). Additionally, an HOCl solution was added to have a free chlorine concentration of 0.20 and 0.28 mg/L, for the groundwater and surface water samples, respectively. In general, there was detachment toward the bulk water phase as indicated by the slopes of the regression models being higher than zero. However, no significant biofilm detachment was observed between the untreated controls and the adjusted waters (Fig. 3, Table A4). Besides, our drinking water setup, and distribution systems in general, demonstrated resilience (36). The pH was restored after 4 h, and the free chlorine concentration was below the detection limit (<0.05 mg Cl_2_/L) after 2 h of recirculation (data not shown). Similarly, the research of Trihn et al. (5) observed the role of biofilms in free chlorine decay.

However, the implementation of the results should be handled with care, as only the short effect (i.e., 8 h) of the operational changes was investigated. Continuous pH changes could alter the electrostatic interactions between materials and microorganisms and between microorganisms (8, 82). Former studies have examined the long-term effect of chlorine on biofilms, mentioning that small increases in chlorine concentrations could lead to a decrease in culturable biofilm bacteria and EPS production (43, 53). Furthermore, in our study, biofilms were grown on glass rings, whereas in a full-scale distribution network aged biofilms on iron or plastic piping materials are used, which are more susceptible toward corrosive water. For example, biofilms attached to stainless steel compound pipes are more sensitive to flushing than those attached to ductile cast iron pipes (56).

### Mature biofilms are susceptible toward the invasion of *Serratia fonticola*

In the next part, we added a GFP-expressing coliform, *S. fonticola* (i.e., ±100 cells/100 mL), into the treated groundwater and surface water samples to investigate invasion onto the corresponding biofilms. Water was recirculated over the biofilm monitor for 4 days, and *S. fonticola* was followed using selective media (Table 4). Our results indicated that the coliform was unable to survive longer than 24 h in the bulk waters, possibly because of the oligotrophic drinking water environment or competition with the resident drinking water community (94–97). However, after 7 days, the coliform was detected in the biofilm samples (Fig. 4; Table 4). Since confocal microscopy has limitations (e.g., operator dependent) and bleaching of the fluorescent protein was observed, the results were validated using qPCR based on the detection of the fluorescent gene, which was incorporated into the genome of the coliform. In the first invasion experiment, the gene copies were below the limit of quantification, but in the second invasion experiment, detection with qPCR was achieved (Table 4). This variation could be attributed to the sampling day (after 7 days instead of 4 days), inoculum concentration, or more favorable invasion circumstances. Additionally, we observed a higher abundance of gene copies/cm² in the treated groundwater biofilm; however, the underlying cause for this observation remains unclear based on the findings of this study. Broadly, adhesion and invasion onto a drinking water biofilm depend on nutrient availability, surrounding microorganisms, local hydrodynamics, and biofilm architecture (82, 95, 98). Further research is needed to understand the influence of these factors, as well as to investigate the importance of other potential variables identified in this study (e.g., sampling day and/or inoculum concentration).

Previous researchers already showed that biofilms could be a reservoir for fecal indicators and pathogens (8, 10). For example, Kilb et al. (99) detected coliforms on rubber-coated valves from distribution networks. Pathogens such as *Pseudomonas aeruginosa* were able to persist in drinking water biofilms after spiking through bulk water samples (12). However, in comparison with the microbial drinking water legislation (i.e., absence in 250 and 100 mL for *P. aeruginosa* and total coliforms, respectively) and the observed concentrations in practice (i.e., 200 cells/100 mL), high spike concentrations were used (i.e., 10^5^–10^7^ cells/mL) in former studies (12, 13, 15–17, 95). Here, in this study, we demonstrated that even low concentrations of coliforms (±100 cells/100 mL) can attach and get established in mature drinking water biofilms. We explicitly chose to use low concentrations as they are relevant for the full-scale practice, where the bigger volumes and flow rates in the DWDS might get contaminated by for example groundwater intrusion after pipe burst, or rain-, river-, or wastewater contamination due to wrong connections (100, 101). Furthermore, it is important to notice that *S. fonticola* was able to settle in two significantly different biofilms regarding cell density and community composition (Fig. 1; Tables 3 and 4). As mentioned before, both biofilms were characterized as mature or quasi-stationary. The concept of quasi-stationary phase was introduced by Boe-Hansen et al. (80), who argue that a true stationary phase is never reached in biofilms due to continuous selection influenced by small changes in environmental conditions. This suggests that certain microcolonies within biofilms may be more susceptible at specific moments.

Water stagnation, changes in flow rates, and flushing after water works can disrupt biofilms, potentially releasing coliforms, and other unwanted microorganisms into the water phase, leading to contamination and associated health concerns (51, 56, 92). Moreover, detached coliforms may settle elsewhere in the DWDS. Despite these risks, further research about biofilm detachment and effective mitigation strategies for preventing the establishment of unwanted microorganisms is necessary to understand the occurrence of contaminations in distribution networks. Favere et al. (94) propose that pathogens and indicator organisms are considered to be r-strategist (high growth rate at high nutrient concentrations), whereas the naturally drinking water community consist out of K-strategist (high substrate affinity). When producing biostable water through nutrient limitation, the bacterial community is directed toward K-strategists, consequently restricting the survival of the r-strategist. In addition, previous research have indicated that by depleting nutrients (such as carbon, nitrogen, or oxygen), both the biological activity in the water and biofilm as well as the production of EPS and subsequent biofilm adhesion, can be reduced (45, 102).

### Conclusion

In conclusion, we have used a biofilm monitor consisting of glass rings to study drinking water biofilms. More specifically, we investigated the response of the biofilm microbiome toward limited operational variations in pH and free chlorine concentration, and the ability of the coliform, *S. fonticola*, to settle onto these biofilms. Two mature drinking water biofilms were characterized using several techniques and it was shown that they were significantly different from each other regarding cell density and community composition. To summarize, biofilms resulting from treated groundwater had a 10 times higher bacterial cell density, ATP content, biofilm thickness, and DAPI-stained biomass concentration than biofilms resulting from treated chlorinated surface water. Next, it was observed that the biofilms remained resilient when applying limited changes that are seen in the full-scale drinking water network. Finally, *S. fonticola*, spiked at low concentrations through the bulk water phase, demonstrated the ability to attach and get established within the mature biofilms, highlighting the potential for biofilms to act as reservoirs for unwanted microorganisms in drinking water distribution systems.

## Data Availability

The data sets presented in this study can be found in online repositories. The names of the repository/repositories and accession number(s) can be found below: https://github.com/waegenaarfien/2023_Unwanted-coliforms-can-hide-in-mature-drinking-water-biofilms.git, https://www.ncbi.nlm.nih.gov/bioproject/PRJNA1015597.

## SUPPLEMENTAL MATERIAL

Supplemental material aem.00042-24-s0001.docx Supplemental methods, Figures SA1 to SA13, and Tables SA1 to SA6.

ASM does not own the copyrights to Supplemental Material that may be linked to, or accessed through, an article. The authors have granted ASM a non-exclusive, world-wide license to publish the Supplemental Material files. Please contact the corresponding author directly for reuse.
